# Biochemical composition of tomato fruits of various colors

**DOI:** 10.18699/VJ21.058

**Published:** 2021-09

**Authors:** A.B. Kurina, A.E. Solovieva, I.A. Khrapalova, A.M. Artemyeva

**Affiliations:** Federal Research Center the N.I. Vavilov All-Russian Institute of Plant Genetic Resources (VIR), St. Petersburg, Russia; Federal Research Center the N.I. Vavilov All-Russian Institute of Plant Genetic Resources (VIR), St. Petersburg, Russia; Federal Research Center the N.I. Vavilov All-Russian Institute of Plant Genetic Resources (VIR), St. Petersburg, Russia; Federal Research Center the N.I. Vavilov All-Russian Institute of Plant Genetic Resources (VIR), St. Petersburg, Russia

**Keywords:** tomato;, fruit color, biochemical compounds, pigments, anthocyanins, томат, окраска плодов, биохимические соединения, пигменты, антоцианы

## Abstract

Tomato (*Lycopersicon esculentum* Mill.) is an economically important and widely cultivated vegetable crop that is consumed both fresh and processed. The nutritional value of tomato fruits is related to the content of carotenoids, polyphenols, sugars, organic acids, minerals and vitamins. Currently, there is a growing interest in the qualitative and quantitative increase in the content of health-promoting compounds in tomato fruits. VIR Lycopersicon (Tourn.) Mill. genetic resources collection includes 7678 accessions of one cultivated and nine wild species, which in turn provides ample opportunities for searching for information on the variability of the content of biologically active substances and searching for sources with a high content of them in the gene pool. Our work presents the results of the study of 70 accessions of cultivated and wild tomato on the main biochemical characteristics: the content of dry matter, ascorbic acid, sugars, carotenoids, chlorophylls and anthocyanins. As the basis for the selection of accessions for the study, accessions with various colors of fruits, including new accessions with varying content of anthocyanin, were taken. As a result of this study, the amplitude of variability in the content of dry matter (3.72–8.88 and 9.62–11.33 %), sugars (1.50–5.65 and 2.20–2.70 %), ascorbic acid (12.40–35.56 and 23.62– 28.14 mg/100 g), titratable acidity (0.14–0.46 and 0.33–0.48 %), chlorophylls (0.14–5.11 and 2.95–4.57 mg/100 g), carotenoids (0.97–99.86 and 1.03–10.06 mg/100 g) and anthocyanins (3.00–588.86 and 84.31–152.71 mg/100 g) in the fruits of cultivated and wild tomatoes, respectively, was determined. We have determined correlations between the content of dry matter and monosaccharides (r = 0.40, p ≤ 0.05), total sugars (r = 0.37, p ≤ 0.05) and ascorbic acid (r = 0.32, p ≤ 0.05); the content of ascorbic acid and carotenoids (r = 0.25, p ≤ 0.05). A high dependence of the content of chlorophyll a and b among themselves (r = 0.89, p ≤ 0.05), as well as between the content of chlorophyll b and anthocyanins (r = 0.47, p ≤ 0.05), the content of β-carotene (r = 0.26, p ≤ 0.05) and the content of monosaccharides (r = –0.29, p ≤ 0.05) has been noted. We have identif ied tomato accessions with a high content of individual chemical substances, as well as with a complex of traits that can be used as sources in breeding for a high content of dry matter, sugars, ascorbic acid, pigments and anthocyanins.

## Abstract (ru)

Томат (*Lycopersicon esculentum* Mill.) – экономически важная и широко возделываемая овощная культура, потребляется как в свежем, так и в переработанном виде. Пищевая ценность плодов томата связа- на с содержанием в них каротиноидов, полифенолов, растворимых сахаров, органических кислот, минера- лов и витаминов. В настоящее время растет интерес к качественному и количественному увеличению содер- жания полезных для здоровья соединений в плодах томата. Коллекция генетических ресурсов Lycopersicon (Tourn.) Mill. Всероссийского института генетических ресурсов растений им. Н.И. Вавилова (ВИР) включает 7678 образцов одного культурного и девяти диких видов, что представляет широкие возможности для поис- ка сведений об изменчивости содержания биологически активных веществ и отбора в генофонде источни- ков с высоким их содержанием. В нашей работе приведены результаты изучения 70 образцов культурного и дикорастущего томата по основным биохимическим признакам – содержанию сухого вещества, аскорби- новой кислоты, сахаров, каротинов, хлорофиллов и антоцианов. Для изучения взяты образцы с разнообраз- ной окраской плодов, включая новые образцы с различным содержанием антоциана. В результате исследо- вания определена амплитуда изменчивости содержания сухих веществ (3.72–8.88 и 9.62–11.33 %), сахаров (1.50–5.65 и 2.20–2.70 %), аскорбиновой кислоты (12.40– 35.56 и 23.62–28.14 мг/100 г), титруемой кислотности (0.14–0.46 и 0.33–0.48 %), хлорофиллов (0.14–5.11 и 2.95–4.57 мг/100 г), общих каротиноидов (0.97–99.86 и 1.03–10.06 мг/100 г) и антоцианов (3.00–588.86 и 84.31–152.71 мг/100 г) в плодах культурного и дикорастуще- го томата соответственно. Определены корреляционные связи между содержанием сухих веществ и сахаридов (r = 0.40, p ≤ 0.05), суммы сахаров (r = 0.37, p ≤ 0.05) и аскорбиновой кислоты (r = 0.32, p ≤ 0.05), содержанием аскорбиновой кислоты и каротиноидов (r = 0.25, p ≤ 0.05). Выявлена высокая зависимость со- держания хлорофиллов a и b между собой (r = 0.89, p ≤ 0.05), а также зависимость средней степени между содержанием хлорофилла b и антоцианов (r = 0.47, p ≤ 0.05), содержанием β-каротина (r = 0.26, p ≤ 0.05) и содержанием моносахаридов (r = –0.29, p ≤ 0.05). Выделены образцы томата с высоким содержанием отдельных химических веществ, а также по комплексу признаков, которые могут быть использованы в качестве источников в селекции на повышенное содержание сухого вещества, сахаров, аскорбиновой кислоты, пиг- ментов и антоцианов.

## Introduction

The beneficial properties of vegetables are associated with the presence of various compounds in them – phytochemicals beneficial to human health. Vegetable crops are the main source of natural antioxidants that have chemoprotective and anti-cancer effects (Zanfini et al., 2010; Chandra, Ramalingam, 2011).

Tomato (**Lycopersicon esculentum** Mill. = syn. Solanum lycopersicum L.) is an economically important crop that ranks first among vegetable crops in terms of cultivation area in the world, and is consumed both fresh and processed. About 182.3 million tons of tomato fruits are grown on 4.85 million hectares in the world annually (FAOSTAT, 2019). Asia accounts for 61.1 % of world production, Europe, America and Africa – 13.5, 13.4 and 11.8 % of the total harvest, respectively. Tomato consumption is concentrated in China, India, North Africa, the Middle East, the USA and Brazil, where it ranges from 61.9 to 198.9 kg per capita (FAOSTAT, 2019).

Tomato was introduced to Europe from Central and Southwest America in the 16th century. It was originally used as an ornamental plant, and then gradually became an important crop in human nutrition (Peralta, Spooner, 2007). As a result of domestication in the world, several different groups of tomato cultivars have been bred, differing in the size, shape and color of the fruits (Bhattarai et al., 2018). Much of the genetic variation was lost during domestication (Bai, Lindhout, 2007), and selection for new productivity traits had a negative impact on several other important traits, such as stress tolerance and fruit quality (Tanksley, 2004; Gascuel et al., 2017).

The nutraceutical value of tomato fruits is explained by the content of carotenoids, polyphenols, soluble sugars, organic acids, minerals and vitamins, especially vitamin C and E (Leiva-Brondo et al., 2012; Raiola et al., 2015; Martí et al., 2016), as well as volatile compounds (Wang, Seymour, 2017). Their antioxidant capacity depends on both lipophilic (carotenoids and vitamin E) and hydrophilic (vitamin C and phenolic compounds) fractions (Ilić et al., 2009). Tomato carotenoids are the main source of lycopene in the human diet (Viuda-Martos et al., 2014). Anthocyanins are not usually found in tomatoes, but flavonols (mainly quercetin, myricetin, and kaempferol) and flavanones (naregenin) have been found (Scarano et al., 2018). Bioactive compounds of tomato fruits have a wide range of physiological properties, including antiinflammatory, antiallergenic, antimicrobial, vasodilating, antithrombotic, cardioprotective and antioxidant effects (Martí et al., 2016; Mozos et al., 2018). Epidemiological evidence suggests that consumption of tomatoes and tomato products is associated with a reduced risk of prostate cancer and other chronic diseases (Campbell et al., 2004; Zanfini et al., 2010; Wei, Giovannucci, 2012; Friedman, 2013).

Currently, there is a growing interest in the qualitative and quantitative increase in the content of healthy compounds in tomato fruits in order to further increase the nutraceutical potential of the crop. Modern biochemical research is aimed at identifying and quantifying the components of plant materials, as well as determining their biological activity. Such data are needed, among other things, for the development of beneficial nutritional and nutraceutical supplements.

VIR Lycopersicon (Tourn.) Mill. worldwide collection includes 7678 accessions of one cultivated and nine wild species (according to C.M. Rick (1959)). Tomato (*L. esculentum* Mill.) has 6536 varietal and 1505 hybrid populations (F3–F5). The first tomato accessions entered in the collection in 1922 as a result of the expedition of N.I. Vavilov and S.M. Bukasov to the USA and Canada. These were stem indeterminate forms with different colors of the fruit, relevant for use in breeding until now. Then the collection was expanded with accessions of various types of growth and development and morphological features from 95 countries all over the world, that is, the widest crop diversity for various uses and sources for breeding is concentrated in it. The collection continues to grow. Currently, much attention is paid to the involvement in the collection of competitive accessions with unconventional fruit color: yellow, orange, pink, crimson, green, brown, purple, “black”, characterized by a high content of biologically active flavonoids and pigments.

The modern structure of the VIR tomato collection: accessions of wild species – 196; primitive forms – 371; landraces – 551; breeding cultivars – 4188; hybrids – 1511; mutant forms – 49; self-pollinated lines – 118; genetic sources with identified genes – 278; donors – 17 accessions.

The purpose of this work is to conduct a comparative assessment of tomato accessions with different fruit colors from the VIR collection in terms of biochemical composition. The main task was to determine the content of the main chemical compounds – dry matter, ascorbic acid, sugars, chlorophylls, carotenoids and anthocyanins – in various tomato accessions from the VIR collection.

## Materials and methods

The material for the study included breeding cultivars and hybrids of different time of creation of cultivated and wild tomatoes from the VIR collection (70 accessions in total), differing in many phenological and morphological characteris tics: the duration of the vegetative period, the type of growth, height of inflorescences formation, the number and type of inflorescences, flower features, fruit formation, shape, size, surface and internal structure of the fruit. Accessions with various colors of fruits were taken for research, including new accessions with different contents of anthocyanin. The studied tomato accessions represented a wide range of colors of fruits in biological ripeness: green-purple, green-yellow, yellow, yellow-orange, yellow-purple, orange, orange-red, red, pink, crimson, red-brown and purple-red, both with a uniform color and with the presence of yellow or green stripes (Table 1).

**Table 1.**
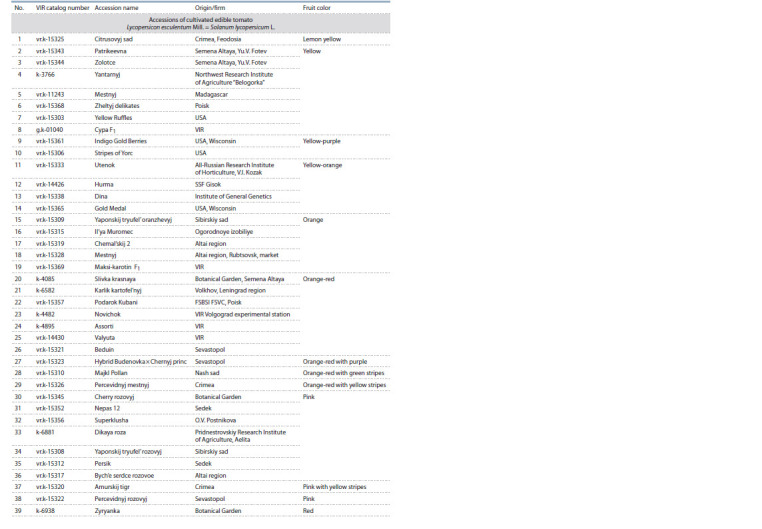
List of the studied tomato accessions

**Table 1(end).**
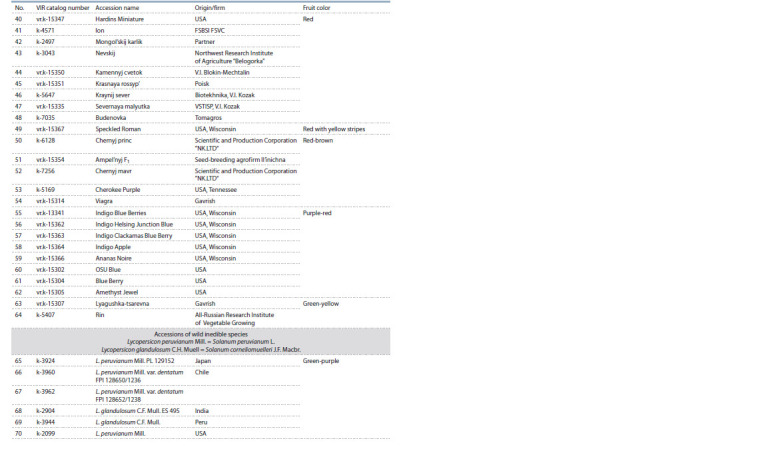
List of the studied tomato accessions(end)

Tomato accessions were grown at the VIR Pushkin and Pavlovsk Laboratories (St. Petersburg, Northwest Russia) in a glass greenhouse in March-October of 2019–2020. The temperature was 22–30 °C during the day and 16–22 °C at night. The plants were grown only under natural light. Daylight duration varied from 11.5 h in March and September to 18.6 h in the third decade of June. Illumination varied from 4 to 10 thousand lx/m2, depending on the growing season. The plants were grown in AgroBalt peat substrate, completely filled with mineral fertilizers. 3–9 plants were placed per 1 m2, depending on the plant habit. Generally accepted in the Northwest region agricultural technologies included the garter and the formation of plants, taking into account the specific conditions of the winter shelving greenhouse.

Phenological and morphological descriptions were carried out according to the “International CMEA Classifier of the Genus *Lycopersicon* Tourn.” (1986), “Descriptors Tomato (Lycopersicon spp.) IPGRI” (1996) and “Tomato – UPOV (*Solanum lycopersicum* L.)” (2012).

**Biochemical analysis** was carried out in the VIR Laboratory of Biochemistry and Molecular Biology in the Biological Ripeness of the Fruits. The study took 1/2 part of at least five fruits of each accession, in two replications. The analysis and processing of the material were carried out according to the VIR methods (Ermakov et al., 1987): the dry matter content was measured by a gravimetric method; sugars – by the Bertrand’s method; total (titratable) acidity – by titrating with 0.1 n of alkali, calculated as malic acid; ascorbic acid – by the method of direct extraction from plants with 1 % hydrochloric acid, followed by titration with 2,6-dichloroindophinol (Tillman’s reagent); carotenoids and chlorophylls were isolated with 100 % acetone and their absorption was measured on an Ultrospec II spectrophotometer at different wavelengths (nm): 645, 662 – for chlorophylls a and b, 440 – for carotenoids, 454 – for carotenes (total carotenes determined by paper chromatography), 454 – for β-carotene, 503 – for lycopene. Anthocyanins were extracted by 1% hydrochloric acid, then measured by spectrophotometry at 510 nm wavelength, in terms of cyanidin-3,5-diglycoside (453 nm), with a correction for the content of the green pigment at 657 nm. All data are presented on raw material.

**Statistical analysis.** Descriptive statistics (mean, median, standard error, standard deviation, range of variability) were calculated for all biochemical parameters to assess the genetic diversity of tomatoes. Data analysis was performed using the STATISTICA v.12.0 software (StatSoft Inc., USA). Data testing for normality of distribution was performed using the Shapiro–Wilk test and the quantile-quantile plot (QQ Plot). The mean values of the data with normal distribution were compared using one-way analysis of variance (ANOVA); Pearson’s correlation coefficient was used for correlation analysis. Data with a distribution other than normal were compared using the Kruskal–Wallis test, and correlation analysis was compared using the Spearman’s rank correlation coefficient. Cluster analysis was performed using the UPGMA method in the PAST program (Hammer et al., 2001).

## Results and discussion

As a result of studying the most important indicators of the biochemical composition of tomato fruits, the large differences between the studied accessions were established.

**Dry matter content**

One of the most important indicators of the quality of tomato fruits and their technological properties is the dry matter content. The dry matter content in the fruits of cultivated tomato was in the range of 3.72–8.88 % (Cv = 14.7 %), in the fruits of wild species – 9.62–11.33 % (Cv = 6.2 %) (Table 2). Fruits with a high concentration of dry substances taste good, give a higher yield during processing, and have better transportability and keeping quality during storage. On average, the red-brown accessions accumulated more dry matter (6.46 %) than the rest. A high content of dry matter (more than 7.00 %) was noted in the accessions Slivka krasnaya, Ampel’nyj F1 and Patrikeevna. Among the accessions of wild species, the largest amount of dry matter in fruits was accumulated by accessions of *L. peruvianum*: 10.25–11.33 %.

In our study, we found weak positive correlations in cultivated tomato accessions between the content of dry matter and monosaccharides (r = 0.40, p ≤ 0.05), the amount of sugars (r = 0.37, p ≤ 0.05) and ascorbic acid (r = 0.32, p ≤ 0.05). Thus, an increase in the amount of these biochemical characteristics in fruits will have little effect on an increase in the other three indicators.

Our results on the dry matter content are consistent with the results of other studies (Gupta et al., 2011; Nour et al., 2013; Kondratyeva, Engalychev, 2019; Ignatova et al., 2020), which reported on the dry matter content in tomato fruits within 5.55–8.80 %.

**Sugar content**

Most of the dry matter in tomato fruits is carbohydrates, the main of which are soluble sugars. In our study, the total sugar content in cultivated forms was 1.50–5.65 % (Cv = 26.2 %), in accessions of wild species – 2.20–2.70 % (Cv = 6.4 %) (see Table 2). The high variability of the sugar content in cultivated tomato is associated with both genetic characteristics and growing conditions. Soluble monosaccharides are represented by glucose and fructose in tomato fruits. The average content of monosaccharides was 2.84 % in cultivated and 1.98 % in wild tomatoes. The accessions Superklusha and Patrikeevna showed a high content of total sugars, including monosaccharides – 5.35 and 5.65 %, respectively.

Oligosaccharides in tomato fruits are mainly represented by the disaccharide sucrose. The content of disaccharides in the studied accessions was low and averaged 0.2–0.4 % in both cultivated and wild forms. Accessions Kamennyj cvetok, Zolotce and Dikaya roza contained more than 1.2 % disaccharides in fruits.

Several studies have shown that green fruits of wild tomato species accumulate mainly sucrose, while fruits of cultivated tomato accumulate glucose and fructose (Stommel, 1992; Beckles et al., 2012). In our study, wild tomato fruits accumulated more monosaccharides than disaccharides, which is possibly related to the growing conditions. The Leningrad region is characterized by low insolation, which is possibly the reason for the low accumulation of disaccharides.

The sugar content in tomato fruits varied in the range of 2.81–4.41 % in the study of A.V. Kuzyomensky (2004), the sugar content was in the range of 2.12–6.00 % in F1 hybrids (Ignatova et al., 2020).

**acidity and sugar-acid index**

The titratable acidity in fruits of cultivated tomato accessions varies within 0.14–0.46 % (Cv = 28.4 %) with an average content of 0.28 %, in wild tomatoes – 0.33–0.48 % (Cv = 15.0 %) with an average content of 0.40 %. A low content of titratable acids (less than 0.19 %) was observed in tomato accessions: Karlik kartofel’nyj, Utenok, Yantarnyj, Gold Medal and Yellow Ruffles. A high content (more than 0.40 %) was noted in tomato accessions with pink, orange, orange-red and yellowpurple color of fruits: Amurskij tigr, Bych’e serdce rozovoe, Stripes of Yorc, Yaponskij tryufel’ oranzhevyj and rozovyj, Valyuta. High acidity in wild tomato species (0.48 %) was noted in two accessions: L. glandulosum (k-3944, Peru) and *L. peruvianum* (k-2099, USA).

Similar results on the level of titratable acidity were obtained in other studies. In R.V. Nour et al. (2013) and J. Owusu et al. (2012) studies titratable acidity varied from 0.10 to 0.41 %.

The taste of the fruit is determined by the index of sugar to acid. It has been proven that this indicator changes depending on soil and climatic conditions, cultivation techniques and varietal characteristics of the crop, as well as the degree of fruit ripeness (Kondratyeva, Pavlov, 2009). The index of sugar to acid is an indicator of the quality of the fruit: the higher it is, the tastier the product.

**Fig. 1.**
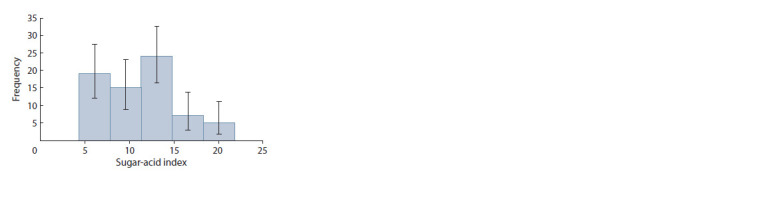
Distribution of tomato accessions by sugar-acid index.

It was found that all accessions had a different sugar-acid index. The level of sugar-acid index in cultivated tomatoes ranged from 4.41–21.80 (Cv = 34.1 %), in wild ones – 4.63–7.01 % (Cv = 14.2 %). Tomato accessions were divided into six statistically significant groups of the sugar-acid index (Fig. 1). The first group included 16 tomato accessions, which were characterized by a low index – 4.4–7.3. This group included all accessions of wild tomato, as well as accessions of cultivated tomato with yellow (Mestnyj), yellow-purple (Stripes of Yorc), orange-red (Valyuta), orange (Yaponskij tryufel’ oranzhevyj), pink (Yaponskij tryufel’ rozovyj, Amurskij tigr), red (Severnaya malyutka, Kraynij sever) and greenyellow (Rin) color of fruits. The second (7.3–10.2), third (10.2–13.1) and fourth (13.1–16.0) groups included 14–17 accessions with different fruit colors. The fifth and sixth groups included accessions with a high index: 16.0–21.8. These groups were represented by accessions with yellow (Patrikeevna), yellow-orange (Dina, Gold Medal), pink (Dikaya roza), red (Zyryanka), orange-red (Karlik kartofel’nyj), purple-red (Indigo Helsing Junction Blue, Amethyst Jewel) and red-brown (Chernyj princ) fruit color.

**Ascorbic acid content**

The nutritional value of tomato fruits is determined, first of all, by the high content of vitamins, among which ascorbic acid (vitamin C) occupies one of the first places. The content of ascorbic acid in the analyzed fruits of cultivated tomato varied from 12.40 to 35.56 mg/100 g (Cv = 24.6 %) with an average content of 20.78 mg/100 g, in wild ones – from 23.62 to 28.14 mg/100 g (Cv = 6.0 %) with an average content of 26.22 mg/100 g. A high content of ascorbic acid (more than 30 mg/100 g) was found in the accessions Utenok, Amethyst Jewel, Yaponskij tryufel’ rozovyj and oranzhevyj. A weak correlation between the content of ascorbic acid with a dry matter content (r = 0.32, p ≤ 0.05) and carotenoids (r = 0.25, p ≤ 0.05) was found in the studied accessions. R.V. Nour et al. (2013) found significant differences in the content of ascorbic acid in different tomato cultivars: 91.9–329.7 mg · kg^–1^. R.A. Dar and J.P. Sharma (2011) found ascorbic acid content in the range of 197.7 to 378 mg · kg^–1^ FW, Harish et al. (2012) – within 20.23–29.32 mg/100 g.

Thus, our results are partially consistent with the already available results and also expand the range of variability of the content of ascorbic acid in tomato fruits.

**Chlorophyll content**

The amount of pigments and their ratio significantly affect the metabolism of plants and can differ depending on the species or cultivar of the plant, as well as on the phase of its ontogenesis (Belova et al., 2012).

Chlorophyll is found in large quantities in unripe tomato fruits; in the process of ripening, it is destroyed. During the ripening of tomatoes, the degradation of chlorophyll happens with the biosynthesis and accumulation of carotenoids at the same time; both processes are responsible for the change of fruit color.

**Fig. 2.**
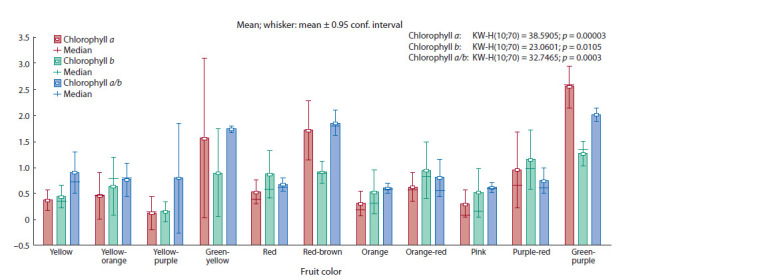
Variability of tomato accessions with different fruit colors in terms of chlorophyll content.

In our study, the total chlorophyll content in cultivated tomatoes was in the range of 0.14–5.11 mg/100 g, in wild ones – 2.95–4.57 mg/100 g (see Table 2). Tomato accessions with different fruit colors differed in the content of chlorophyll a and b (Fig. 2).

**Table 2.**
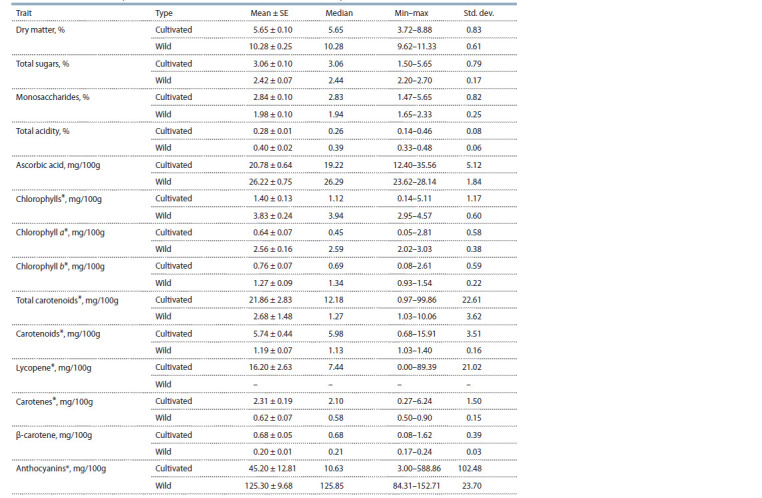
Parameters of descriptive statistics of cultivated and wild tomato accessions by biochemical characteristics * The data have abnormal distribution.

In addition to the total chlorophyll content, the adaptability of plants to a certain lighting regime is also manifested in the qualitative composition of pigments. In our study, as expected, the largest amount of chlorophyll a in fruits was accumulated by accessions of wild tomato. A high content was found in accessions of cultivated forms with green-yellow (1.45–1.69 mg/100 g), red-brown (1.25–2.32 mg/100 g) fruit color and in several accessions with purple-red fruit color: Blue Berry (vr.k-15304) – 1.53 mg/100 g and Indigo Clackamas Blue Berry (vr.k-15363) – 2.81 mg/100 g. The other accessions contained not more than 1.00 mg/100 g of chlorophyll a.

Tomato accessions with yellow, yellow-orange, red, orange, orange-red, pink and purple-red fruit color accumulated more chlorophyll b in the fruit. The highest content (more than 1.00 mg/100 g) was noted in the fruits of wild tomato and in most accessions of cultivated tomato with purple-red color of fruits, as well as some accessions with red: Mongol’skij karlik (1.20 mg/100 g), Nevskij (1.76 mg/100 g), Kraynij sever (2.43 mg/100 g); orange-red: Beduin (1.14 mg/100 g), Podarok Kubani (1.89 mg/100 g), Valyuta (2.61 mg/100 g); and pink: Cherry rozovyj (1.46 mg/100 g), Nepas 12 (1.58 mg/100 g), fruit color.

One of the informative indicators characterizing the potential photochemical activity of fruits is the ratio of chlorophyll a to chlorophyll b (a/b). The possible effect of fruit ripening on the rate of destruction of pigments was reflected in the value of the ratio of the chlorophyll content – a to b. In our study, chlorophyll b prevails in the total chlorophyll pool of cultivated tomatoes. The chlorophyll a/b ratio was in the range of 0.45–2.17 in most cultivated tomato accessions, and in wild ones – 1.85–2.53. All accessions with yellow-green and redbrown fruit color had a chlorophyll a/b ratio more than 1, as well as some accessions with yellow (Zheltyj delikates, Zolotce), red (Zyryanka), orange-red (Novichok, Hybrid Budenovka × Chernyj princ) and purple-red (Blue Berry, Indigo Clackamas Blue Berry) color of the fruit.

In general, accessions with green-purple, green-yellow, red-brown and purple-red coloration in total accumulated more chlorophylls in their fruits – more than 2.40 mg/100 g, accessions with orange-red, red and yellow-orange coloration – within the range of 1.10–1.58 mg/100 g, the total content of chlorophylls in fruits with a different color did not exceed 0.85 mg/100 g.

Thus, it can be assumed that the presence of chlorophylls in tomato fruits with yellow, yellow-orange, orange, orange-red, red and pink coloration is due to the fact that the chlorophyll degradation process was not yet completed and proceeded in parallel with the synthesis of carotenoids, and the prevalence of chlorophyll b indicates that the rate of photosynthesis has already been reduced. The high accumulation of chlorophylls in the fruits of some tomato accessions with red, orange-red and yellow-orange color of the fruit may be associated with the presence of a green spot.

**Carotenoid content**

The diversity of tomato fruit color is the result of mutations in the genes of the carotenoid pathway, which arose as a result of domestication and improvement of varieties, such as yellowf lesh ( r), tangerine ( t), green-f lesh ( gf ), green ripe ( gr), apricot (at ), beta carotene (B), high pigment (hp), old gold (og), and y ( yellow) (Roohanitaziani et al., 2020). Changing the classic red color of tomato, such genes primarily affect its biochemical composition, and especially the content of carotenoids, allowing the creation of varieties with a changed content of these substances (Kuzyomensky, 2004).

The most common carotenoids of red tomato varieties are lycopene (red pigment) and β-carotene (yellow-orange pigment), while lutein, ζ-carotene, neurosporin, and others may also be present in orange and yellow fruits (Khachik et al., 2002). Other identified tomato carotenoids – γ-carotene, phytoene, phytofluen, are found in small amounts (Golubkina et al., 2017).

The pigment complex of the fruits of the studied tomato accessions was characterized by a high content of carotenoids. The total content of carotenoids in the fruits of cultivated tomato was in the range of 0.97–99.86 mg/100 g, with an average content of 21.86 mg/100 g, in wild tomato – 1.03– 10.06 mg/100 g, with average content – 2.68 mg/100 g.

**Fig. 3.**
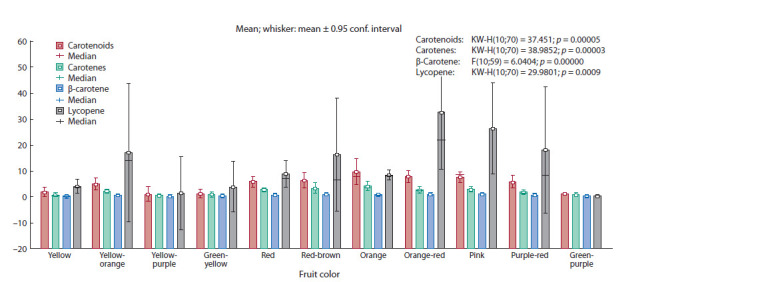
Variability of tomato accessions of various fruit colors in terms of the content of carotenoids.

The variability of the carotene content in tomato accessions was high (Cv = 64.9 %). The average carotene content of cultivated tomato accessions was 2.31 mg/100 g, of which β-carotene was 0.68 mg/100 g (Fig. 3). High carotene content was found in tomato accessions with red-brown (average 3.25 mg/100 g) and orange (4.03 mg/100 g) fruit color, low (less than 0.80 mg/100 g) was found in accessions with yellow, green-yellow, yellow-purple and green-purple color of fruits, the rest contained on average 1.66–2.85 mg/100 g. At the same time, a high content of β-carotene was found in tomato accessions with pink (average 0.89 mg/100 g) and orange-red (0.95 mg/100 g) fruit color, slightly less (0.81–0.82 mg/100 g) in accessions with red-brown and orange colored fruits. Accessions Valyuta (vr.k-14430), Kraynij sever (k-5647), and Novichok (k-4482) contained more than 1.40 mg/100 g of β-carotene.

Lycopene is a non-cyclic β-carotene isomer. The content of lycopene in fruits of cultivated tomato varied from 0.00 to 89.39 mg/100 g; lycopene was not found in fruits of wild tomato (see Table 2). The differences between the accessions in terms of lycopene content are very large, including the differences within the fruit color groups. Accessions with pink and orange-red color of fruits were characterized by a high lycopene content (on average 26.32–32.52 mg/100 g), accessions with green-yellow, yellow and yellow-purple color of the fruit accumulated significantly less (less than 6.5 mg/100 g). Accessions with red and orange color of fruits in our study had similar values for lycopene content – 8.80 and 8.37 mg/100 g, as well as accessions with red-brown, yellow-orange and purple-red color of fruits: 16.12, 17.04 and 18.04 mg/100 g, respectively (see Fig. 3).

Accessions with yellow-orange (Dina and Gold Medal), red-brown (Viagra) and purple-red (OSU Blue) color of the fruit showed a high content of lycopene (for each group: 21.62,38.71, 45.67 and 89.39 mg/100 g, respectively). Accession Zheltyy delikates did not accumulate lycopene in the fruit.

The data on the content of lycopene and β-carotene is very different in the works of other authors. As a result of studying 10 red tomatoes, R.V. Nour et al. (2013) found that the content of lycopene was in the range of 19.7–49.0 mg · kg^–1^, and β-carotene – 6.4–12.8 mg · kg^–1^. After studying 185 tomato accessions, S. Anjum et al. (2020) determined that the content of lycopene was 1.57–23.24 mg · 100 g^–1^, β-carotene – 1.32–7.61 mg · 100 g^–1^. R.S. Pal et al. (2018) reported the content of lycopene in the studied 22 tomato lines in the range of 3.05–9.83 mg/100 g and β-carotene – 4.32–7.31 mg/100 g. In a study by I.Yu. Kondratyeva and N.A. Golubkina (2016), the content of lycopene in tomato accessions with yellow and orange color of the fruit was in the range of 0.0–2.6 mg/100 g, in fruits with red and pink color – 3.3–11.5 mg/100 g, β-carotene – 0.8–6.2 and 0.8–3.1 mg/100 g, respectively.

In our study, the proportion of β-carotene from the total content of carotenes is 25.7–28.4 % in accessions with yellow, yellow-orange and yellow-purple color of fruits, and the proportion of carotenes from carotenoids is 41.5–42.8 %. Thus, we can assume that the remaining carotenoid pigments in these accessions are xanthophylls, including lutein. At the same time, accessions with a yellow-orange color of the fruit accumulated a significant amount of lycopene (on average 17.0 mg/100 g). Accessions with a green-yellow color of the fruit were characterized by a high proportion of carotenes in the carotenoid complex – 71.7 %, but β-carotene averaged only 20.0 %. In accessions with red, red-brown and orange fruit color, the proportion of carotenes was in the range of 42.5–52.0 %, and β-carotene – 20.1–26.3 %, while the accessions with these fruit colors accumulated the greatest amount of carotenes (on average 2.7–4.0 mg/100 g) compared to the rest of the accessions. Accessions with orange-red and pink color of fruits accumulated the greatest amount of lycopene – on average 26.3–32.5 mg/100 g, while the proportion of carotenes was small – 34.5–38.2 %, with a proportion of β-carotene within 31.3–36.1 %. In accessions with a purplered color of the fruit, the proportion of carotenes was 29.0 % with a prevalence of β-carotene.

Thus, we can assume that tomato fruits also contain other carotenoid pigments that were not identified by us – lutein, ζ-carotene, γ-carotene, neurosporin, phytoene, phytofluen and others, which is consistent with the studies of other authors (Khachik et al., 2002; Golubkina et al., 2017).

**Anthocyanin content**

Normally, cultivated tomato plants do not synthesize anthocyanins in fruits. Three loci, Anthocyanin fruit (Aft ), atroviolacium (atv), and Aubergine (Abg), enhance the accumulation of anthocyanins in fruits when they introgress from wild species into cultivated tomatoes (Kendrick et al., 1997; Jones et al., 2003). The atv, Aft, and Abg loci in wild tomato species can contribute to the pigmentation of anthocyanins in fruits, and the atv locus can dramatically increase the amount of anthocyanins in cultivated tomato fruits when it is combined with the Aft or Abg locus (Mes et al., 2008). Most of the anthocyanins present in the fruits of such tomatoes are concentrated in the skin, and almost complete absent in the seeds and pulp (Ooe et al., 2016).

**Fig. 4.**
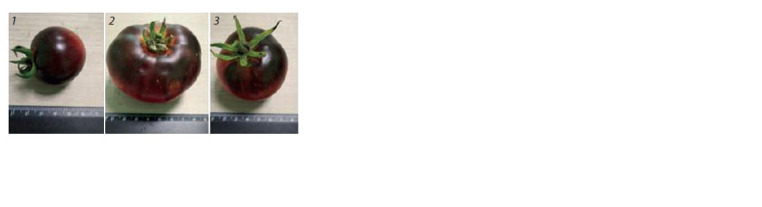
Accessions of tomato high in anthocyanins: Indigo Clackamas Blue Berry (1), Indigo Apple (2), Ananas Noire (3).

In our study, a significant amount of anthocyanins was observed in accessions of cultivated tomato with purplered (32.89–588.86 mg/100 g) and yellow-purple (87.91– 161.22 mg/100 g) fruit color, as well as in wild tomato fruits (84.31–152.71 mg/100 g) (Fig. 4).

In accessions with other fruit colors, anthocyanins were also found, but in much smaller quantities. Fruits with red coloration accumulated anthocyanins on average 14.09 mg/100 g, with yellow, yellow-orange, green-yellow, orange and orangered – within 10.62–11.77 mg/100 g, and accessions with redbrown and pink fruit color – less than 9.0 mg/100 g. Anthocyanin content of 53.3 mg/100 g was found in the Speckled Roman accession with red and yellow stripes.

The correlation analysis revealed a high dependence of the content of chlorophyll a and b among themselves (r = 0.89, p ≤ 0.05), as well as an average positive correlation between the content of chlorophyll b and anthocyanins (r = 0.47, p ≤ 0.05), weak – with the content of β-carotene (r = 0.26, p ≤ 0.05) and weak negative – with the content of monosaccharides (r = –0.29, p ≤ 0.05). Between chlorophyll a and anthocyanin there is also a positive correlation of average degree (r = 0.37, p ≤ 0.05).

C.M. Jones et al. (2003) reported that the amount of anthocyanins in the peel of “blue” tomatoes ranged from 20.6 to 66.5 mg/100 g, in another study the amount of anthocyanins in the peel ranged from 7.79 to 110.79 mg/100 g (Peter et al., 2008). In studies of “purple” tomatoes obtained by the method of genetic engineering, *Del/Ros1, Del/Ros1 ×AtMYB12,* the anthocyanin content is reported to be 5.1 ± 0.5 g · kg^–1^ DW and 1.154 ± 0.011 mg · g–1 FW (Lim et al., 2014; Zhang et al., 2015), and in tomato accessions obtained by breeding *Aft/Aft × atv/atv* – 116.11 mg · 100 g^–1^ FW (Mes et al., 2008), V118 – 50.18 mg · 100 g^–1^ DW (Li et al., 2011), Blue Japan Indigo tomato – 17 mg · g–1 DW (Ooe et al., 2016) and *Aft/Aft × atv/atv × hp2/hp2* – 90.91 mg · 100 g^–1^ FW (Da Silva- Souza et al., 2020). E. Ooe et al. (2016) also reported that “blue” tomato accessions accumulate significant amounts of lycopene.

Thus, our studies of the anthocyanin content in tomato fruits are consistent with previous studies. As a result of the biochemical analysis, we identified tomato accessions by a set of traits that can be used as sources in breeding for a high content of sugars and biologically active substances (Table 3).

**Table 3.**
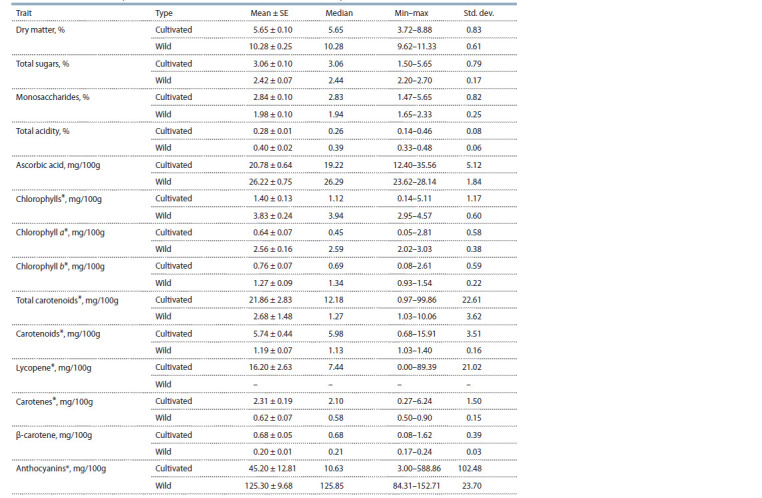
Parameters of descriptive statistics of cultivated and wild tomato accessions by biochemical characteristics * The data have abnormal distribution.

**Cluster analysis**

**Fig. 5.**
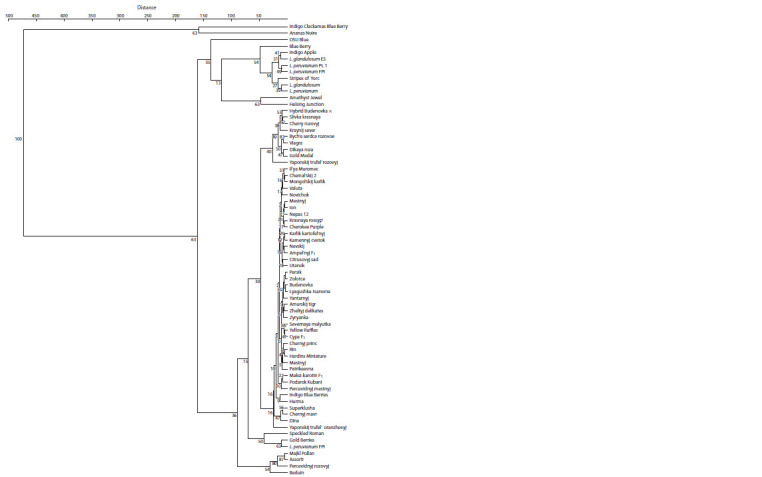
Dendrogram of tomato accessions by basic biochemical parameters. UPGMA method. The numbers indicate the size of the bootstrap; the names of the accessions are given in accordance with Table 1.

A dendrogram was constructed based on the results of cluster analysis of the studied biochemical parameters of tomato accessions (in accordance with Table 2) (Fig. 5). Tomato accessions were divided into two groups, small and large; within the second group, five clusters were identified.

The first cluster included two tomato accessions with a high content of anthocyanins and chlorophylls in fruits: Ananas Noire (430.3 and 2.63 mg/100 g) and Indigo Clackamas Blue Berry (588.9 and 5.11 mg/100 g).

The second cluster is divided into three sub-clusters. The first subcluster is represented by one accession from the USA – OSU Blue, the second – by accessions of wild tomato L. glandulosum (k-2904, k-3944) and *L. peruvianum* (k-2099, k-3924, k-3962), as well as accessions of cultivated tomato Stripes of Yorc with a yellow-purple color of the fruit and Indigo Apple (vr.k-15364) and Blue Berry (vr.k-15304) with a purple-red color of the fruit. The third subcluster included two accessions with a purple-red color of the fruit – Amethyst Jewel and Indigo Helsing Junction Blue. This group of accessions was also characterized by a high content of anthocyanins from 120.4 to 281.3 mg/100 g, and the accessions of the first and third subclusters had a high content of lycopene: 89.4, 16.4 and 11.6 mg/100 g, respectively.

The third cluster included tomato accessions with pink (Dikaya roza, Bych’e serdce rozovoe, Cherry rozovyj, Yaponskij tryufel’ rozovyj), orange-red (Slivka krasnaya, Hybrid Budenovka × Chernyj princ), yellow-orange (Gold Medal), red (Kraynij sever) and red-brown (Viagra) fruit color. These accessions were characterized by a high content of total carotenoids – 44.96 ± 5.97 mg/100 g, of which there were 36.57 ± 6.45 mg/100 g of lycopene, 3.01 ± 1.37 mg/100 g of carotenes, and were low in anthocyanins.

The fourth cluster was the largest; it combined 41 accessions with different fruit colors and was divided into six subclusters. The first subcluster is represented by 16 accessions, mainly with red and orange color of the fruit, which were characterized by the content of carotenes – on average 3.03 ± 1.31 mg/100 g and lycopene – 6.35 ± 1.92 mg/100 g; this group also included several accessions with a high content of chlorophylls. The second subcluster also combined 16 accessions, but mainly with yellow and green-yellow fruit coloration and several with red and pink. These accessions were characterized by a low content of total carotenoids (6.28 ± 2.36 mg/100 g), including lycopene – an average of 3.78 ± 2.50 mg/100 g. The third subcluster is represented by three accessions with orange and orange-red fruit coloration. They were characterized by a high content of chlorophylls – 1.19–2.88 mg/100 g, anthocyanins – 18.41 ± 5.04 mg/100 g and total carotenoids – 17.04 ± 2.22 mg/100 g, of which the content of lycopene was 10.54 ± 0.15 mg/100 g, carotene – 3.56 ± 1.33 mg/100 g. The fourth subcluster is formed by two accessions – Indigo Blue Berries with a purple-red color and Hurma with a yellow-orange color of the fruit. The fifth subcluster is represented by three accessions: Dina, Chernyj mavr and Superklusha with a high content of total carotenoids (on average 25.71 ± 1.59 mg/100 g), of which the content of lycopene was 18.66 ± 3.23 mg/100 g and the content of carotenes was 3.28 ± 1.75 mg/100 g, of which β-carotene content was an average of 0.83 ± 0.38 mg/100 g, and a total sugar content of 3.91 % on average. The sixth subcluster included one accession of the Yaponskij tryufel’ oranzhevyj, which is characterized by a high content of all carotenoids and low chlorophylls, as well as a high content of ascorbic acid and titratable acidity.

The fifth cluster included accession of wild tomato *L. peruvianum* (k-3960) and two accessions of cultivated tomato with yellow-purple (Indigo Gold Berries) and red with yellow stripes (Speckled Roman) fruit color. These accessions had an average content of anthocyanins in fruits – in the range of 53.3–87.9 mg/100 g.

The sixth cluster is represented by four accessions with orange-red (Beduin, Assorti, Majkl Pollan) and pink (Percevidnyj rozovyj) color of the fruit, which were characterized by a high content of lycopene – on average 71.90 ± ± 9.91 mg/100 g.

Thus, the accessions of the first two clusters were characterized by a high content of anthocyanins and chlorophylls in fruits, as well as ascorbic acid. The accessions of the second and fifth clusters were distinguished by a high dry matter content, while the accessions of the third and sixth clusters had a high content of total sugars, total carotenoids, with a predominance of lycopene and β-carotene. The fourth cluster united tomato accessions, on average, with a low content of carotenoids and anthocyanins, but a high content of carotenes. The accessions of the fifth cluster were characterized by an average content of anthocyanins and a low content of carotenoids.

## Conclusion

As a result of this study, it was revealed that tomato accessions from the VIR collection with different fruit colors greatly differ in biochemical composition. We have determined the amplitude of variability of the main biochemical characteristics: dry matter, sugars, ascorbic acid, titratable acidity, pigments and anthocyanins. Correlations were revealed between the content of dry matter and monosaccharides (r = 0.40, p ≤ 0.05), the total sugars (r = 0.37, p ≤ 0.05) and ascorbic acid (r = 0.32, p ≤ 0.05); the content of ascorbic acid and carotenoids (r = 0.25, p ≤ 0.05). A high dependence of the content of chlorophyll a and b among themselves (r = 0.89, p ≤ 0.05), as well as an average positive relationship between the content of chlorophyll b and anthocyanins (r = 0.47, p ≤ 0.05), weak with the content of β-carotene (r = 0.26, p ≤ 0.05) and weak negative with the content of monosaccharides (r = –0.29, p ≤ 0.05) was demonstrated. There was also a moderate positive correlation between the content of chlorophyll a and anthocyanin (r = 0.37, p ≤ 0.05).

It was revealed that accessions with red-brown color of fruits accumulate more dry matter. Accessions with greenpurple, green-yellow, red-brown and purple-red coloration in total accumulate more chlorophylls in fruits – more than 2.40 mg/100 g, accessions with orange-red, red and yelloworange coloration – within 1.10–1.58 mg/100 g. Tomato accessions characterized by a high content of carotene are those with red-brown (average 3.25 mg/100 g) and orange (4.03 mg/100 g) fruit color, whereas accessions with yellow, green-yellow, yellow-purple and green-purple color of fruits – by a low carotene content (less than 0.80 mg/100 g). A high content of β-carotene was found in tomato accessions with pink (average 0.89 mg/100 g) and orange-red (0.95 mg/100 g) fruit color, a lower content (0.81–0.82 mg/100 g) – in accessions with red-brown and orange fruit color.

It was determined that the differences in the content of lycopene between the accessions are very large, including the differences within the fruit color groups. A high content of lycopene was found in accessions with pink and orange-red color of fruits (on average 26.32–32.52 mg/100 g), accessions with green-yellow, yellow and yellow-purple color of the fruit accumulated it much lower – less than 6.5 mg/100 g. A large amount of anthocyanins was contained in tomato accessions with purple-red (32.89–588.86 mg/100 g) and yellow-purple (87.91–161.22 mg/100 g) fruit color, as well as in accessions of wild tomato (84.31–152.71 mg/100 g). Anthocyanins were also found in accessions with different color of fruits, but in much smaller quantities.

We have identified tomato accessions with a high content of both individual chemicals and a complex of traits that can be used as sources in breeding for a high content of dry matter, sugars, ascorbic acid, pigments and anthocyanins.

## Conflict of interest

The authors declare no conflict of interest.
